# Selumetinib-based therapy in uveal melanoma patient-derived xenografts

**DOI:** 10.18632/oncotarget.24670

**Published:** 2018-04-24

**Authors:** Didier Decaudin, Rania El Botty, Béré Diallo, Gerald Massonnet, Justine Fleury, Adnan Naguez, Chloé Raymondie, Emma Davies, Aaron Smith, Joanne Wilson, Colin Howes, Paul D. Smith, Nathalie Cassoux, Sophie Piperno-Neumann, Sergio Roman-Roman, Fariba Némati

**Affiliations:** ^1^ Laboratory of Preclinical Investigation, Department of Translational Research, Institut Curie, PSL University Paris, Paris, France; ^2^ Department of Medical Oncology, Institut Curie, Paris, France; ^3^ IMED Oncology, AstraZeneca, Cambridge, UK; ^4^ Department of Oncological Ophthalmology, Institut Curie, Paris, France; ^5^ Department of Translational Research, Institut Curie, PSL University Paris, Paris, France

**Keywords:** uveal melanoma, patient-derived xenografts, selumetinib, targeted therapies, pharmacodynamics

## Abstract

The prognosis of metastatic uveal melanoma (UM) is among the worst of all human cancers. The identification of near-ubiquitous GNAQ/GNA11 mutations and the activation of MAPK signaling in UM have raised hopes of more effective, targeted therapies, based on MEK inhibition, for example. We evaluated the potential of drug combinations to increase the efficacy of the MEK inhibitor selumetinib (AZD6244, ARRY-142886), in UM cell lines and Patient-Derived Xenografts. We first evaluated the combination of selumetinib and DTIC. We found that DTIC did not improve the *in vitro* or *in vivo* antitumor efficacy of selumetinib, consistent with the outcome of the SUMIT clinical trial assessing the efficacy of this combination in UM. We then tested additional selumetinib combinations with the chemotherapy agent docetaxel, the ERK inhibitor AZ6197, and the mTORC1/2 inhibitor, vistusertib (AZD2014). Combinations of selumetinib with ERK and mTORC1/2 inhibitors appeared to be the most effective in UM PDX models.

## INTRODUCTION

There is a wealth of genetic information characterizing uveal melanoma (UM) biology [1, 2]. Nevertheless, effective treatments for this disease are still lacking, and patients with metastatic UM have a very poor prognosis, among the worst of all human cancers, with a median survival of about 12 months [3], [4]. *GNAQ/11* gene mutations occur in about 85% of UM cases. *GNAQ* and *GNA11* encode small GTPases [5] involved in protein kinase C (PKC) activation. Thus, *GNAQ/11* mutations induce the constitutive activation of the PKC/MAPK pathways involved in oncogenesis [6–8]. The MAPK signaling pathway is upregulated in these tumors, raising the possibility of targeted therapies. Moreover, genotype-dependent anti-tumor effects of MAPK pathway inhibition have been observed, acting at the level of the mitogen-activated protein kinase enzymes MEK1 and MEK2 in preclinical models [8, 9]. There is, therefore, a rationale basis for therapeutic interventions with the MEK inhibitor (MEKi) selumetinib (AZD6244, ARRY-142886). The first phase II clinical trial for selumetinib assessed the efficacy of this drug for uveal melanoma treatment through comparisons with temozolomide and dacarbazine [10]. Progression-free survival and response rates were better in patients with metastatic UM treated with selumetinib than in patients treated with chemotherapy, but overall survival was no higher. The SUMIT phase III trial compared the efficacy of selumetinib with that of temozolomide or dacarbazine, with a view to improving clinical outcomes in patients [11]. The combinations tested did not improve PFS in patients with metastatic uveal melanoma. In the first part of this study, we evaluated the antitumor effect of a combination of selumetinib with dacarbazine. Our results were consistent with the outcome of the SUMIT trial. New therapeutic approaches are, therefore, required, to achieve significant improvements in outcome in patients with metastatic UM.

Several recent studies have evaluated novel combinations of targeted therapies in preclinical models of UM. These combinations have included MAPK, Pi3K, PKC, and/or p53-MDM2 inhibitors [12–15], and various other targets, such as Bcl-2 [16] and c-MET [17], have also been considered. However, none of these treatments has been shown to improve the dismal prognosis and natural course of metastatic UM in patients. One of the key challenges in translational research is the development of relevant preclinical models for the testing of clinical concepts, in so-called ‘co-clinical’ trials [18]. The tolerability of treatments in humans remains one of the most important clinical criteria potentially blocking the development of new treatments. One way of overcoming the risk of tolerance problems is to include drugs that have already been approved, or have at least been through clinical testing, for which the safety margins are well known, in treatment combinations. For metastatic UM, the use of combinations of treatments with different toxicity spectra is another way to prevent intolerance.

Based on all these considerations, we therefore tested various selumetinib-based drug combinations on a panel of UM patient-derived xenografts (PDXs) developed and characterized in our laboratory. These experiments identified combinations that were consistently effective in the models tested. These potentially relevant therapeutic regimens maynow be tested in clinical trials for metastatic UM.

## RESULTS

### A trend towards synergy between selumetinib and DTIC in uveal melanoma cell lines *in vitro*

**Figure 1:**
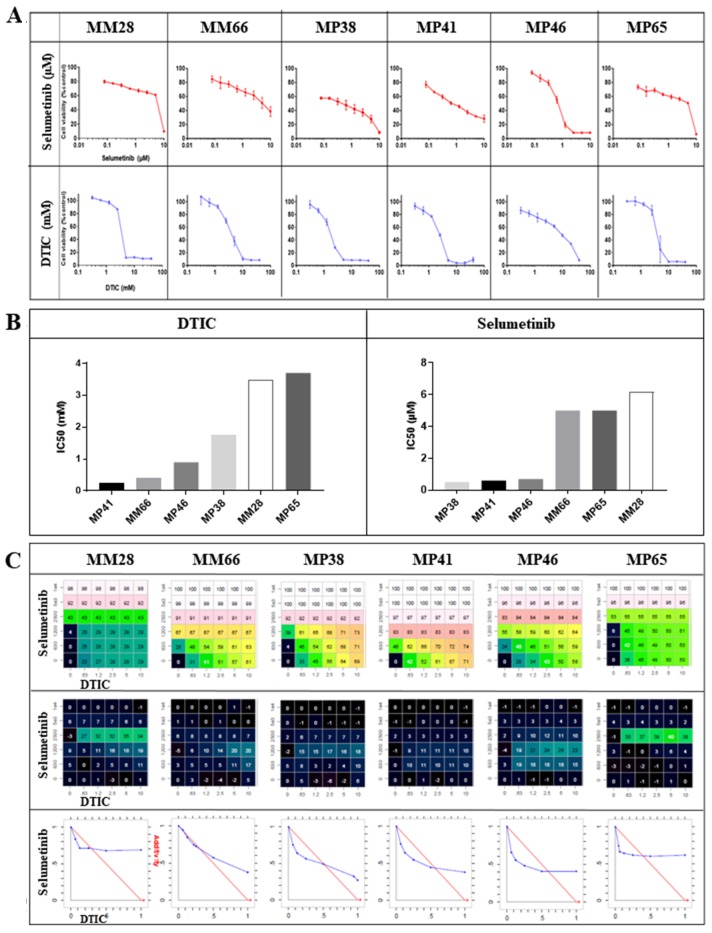
*In vitro* efficacy of selumetinib and DTIC **(A)** Dose-response curves for selumetinib and DTIC in the six UM cell lines. **(B)** IC_50_ values for selumetinib and DTIC in the six UM cell lines. **(C)** Synergy between selumetinib and DTIC: the matrix representing percent growth inhibition *(top panels)*, the matrix with the Loewe Excess results *(middle panels)* and isobolograms *(bottom panels)* are shown. In the isobolograms, the expected additivity line is shown in red and the experimental data are shown in blue.

We first investigated the effects of selumetinib and DTIC separately, in six UM cell lines. The dose-response curves for these two drugs are presented in Figure 1A. Three cell lines, MP38, MP41 and MP46, were found to be sensitive to selumetinib, with EC_50_ values of 0.5 to 0.7 μM, whereas the other three cell lines tested, MM28, MP65 and MM66, were relatively resistant, with EC_50_ values of about 5 μM. Two of the resistant cell lines (MM28 and MM66) were derived from liver metastases. The UM cell lines tested displayed some resistance to DTIC, with EC_50_ values greater than 250 μM; Figure 1B).

The data for studies of combinations with selumetinib are summarized in Figure 1C. A weak synergistic effect of the combination was observed in five of the six cell lines. This effect was observed at a concentration of 1.2 mM DTIC in MP38, MP41, and MP46 cells, and at 2.5 mM DTIC in MM28 and MP65 cells, for all concentrations of selumetinib tested. In the case of MM66 cells, an additive effect was observed at DTIC concentrations of 0.63 mM and 1.2 mM.

Overall, these data reveal a non-significant trend towards synergy between selumetinib and DTIC in UM cell lines.

### The efficacy of selumetinib against UM PDXs *in vivo* is not significantly improved by DTIC

**Figure 2:**
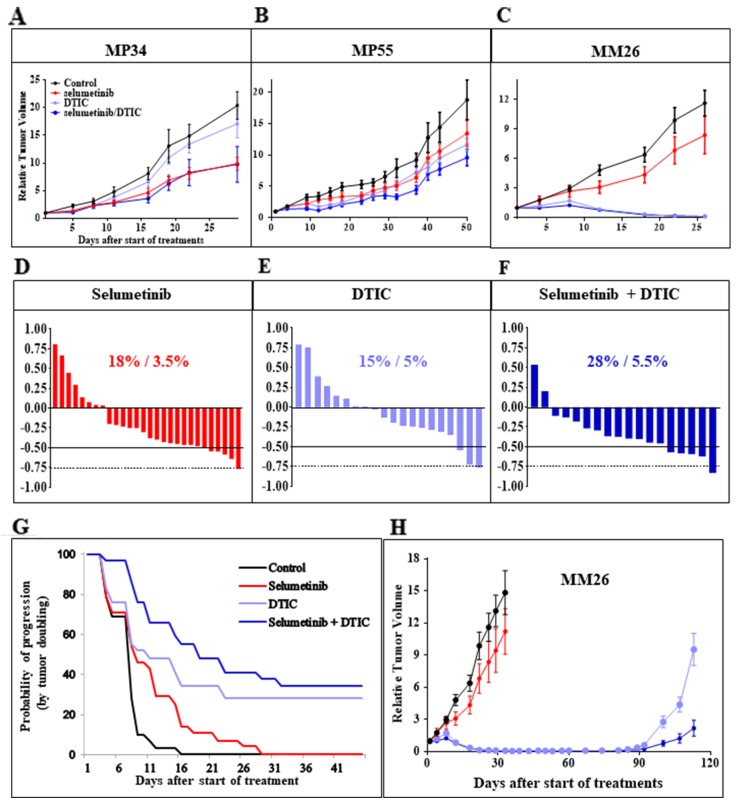
*In vivo* efficacy of selumetinib + DTIC **(A-C)** Tumor growth inhibition obtained with selumetinib with and without DTIC in the three UM PDXs, MP34 (A), MP55 (B), and MM26 (C). Tumor growth was evaluated by plotting mean RTV (relative tumor volume) ± SEM for each group. **(D-F)** Overall responses to selumetinib (D), DTIC (E), and selumetinib + DTIC (F). **(G)** Probability of progression after selumetinib treatment with or without DTIC, taking tumor doubling time into account. **(H)** *In vivo* relapses after DTIC-induced complete remission, for the MM26 UM PDX, with and without selumetinib administration.

We then assessed the efficacy of selumetinib as a single agent in three uveal melanoma PDXs: MP34, MP55, and MM26 (Figure 2). The MP34 PDX displayed significant tumor growth inhibition (TGI; 54%, *p* < 0.02). By contrast, no significant TGI was obtained in the MP55 and MM26 models (Figure 2A–2C). DTIC did not significantly affect tumor growth in the MP34 and MP55 models, but it caused 99% TGI in the MM26 PDX, with five cases of complete remission (CR) among the nine mice treated (55%). A relapse occurred in one of these responder mice relapsed after 11 days of CR (Figure 2H). The combination of selumetinib and DTIC was not significantly more effective than either of these drugs used in monotherapy in MP34 and MP55 cells. In MM26 cells, the combination induced CR in five of the nine animals (55%), with no relapses. The combination did not significantly modify the overall response rate (ORR; Figure 2D–2F) or the probability of progression when tumor doubling time was taken into account (Figure 2G). The concomitant administration of DTIC in mice without tumor grafts had no effect on selumetinib pharmacokinetics, as estimated by determining plasma free selumetinib concentrations over a range of time points after six days of treatment (Supplementary Figure 1). Selumetinib concentration in the blood peaked two hours after the last oral intake and was unchanged by DTIC. We can therefore conclude that the absence of synergy between selumetinib and DTIC *in vivo* was not due to changes in selumetinib pharmacokinetics. Overall, the efficacy of the combination of selumetinib and DTIC was no greater than that of single agents.

### Pharmacodynamics (PD) studies of the combination of selumetinib and DTIC

**Figure 3:**
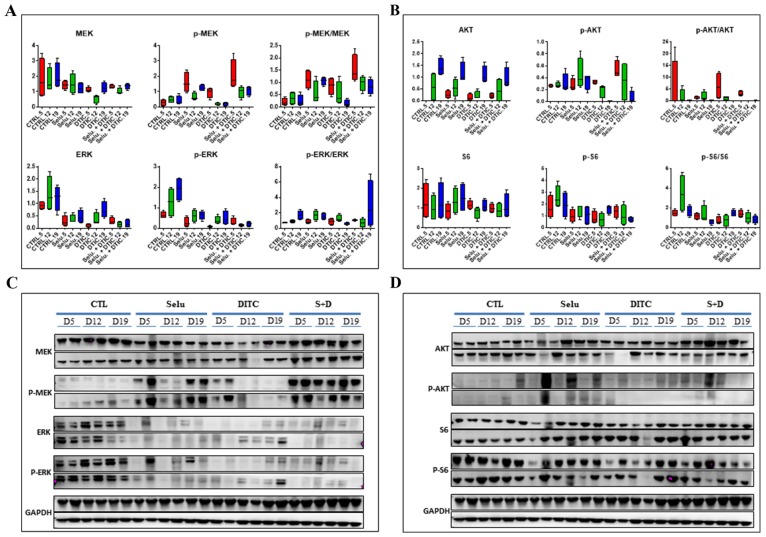
Pharmacodynamic (PD) kinetics study for the combination of selumetinib and DTIC in the MP55 UM PDX **(A)** Evaluation of normalized MAPK protein levels (MEK, p-MEK, ERK, p-ERK) at three different time points after selumetinib, DTIC, or selumetinib + DTIC administration *in vivo*. **(B)** Evaluation of normalized Pi3K protein levels (AKT, p-AKT, S6, p-S6) at three different time points after selumetinib, DTIC, or selumetinib + DTIC administration *in vivo*. **(C)** Western blot of MAPK proteins (MEK, p-MEK, ERK, p-ERK) at three different time points after selumetinib, DTIC, or selumetinib + DTIC administration *in vivo*. **(D)** Western blot of normalized Pi3K protein levels (AKT, p-AKT, S6, p-S6) at three different time points after selumetinib, DTIC, or selumetinib + DTIC administration *in vivo*.

A PD study on the MP55 PDX showed that treatment with selumetinib alone decreased p-ERK expression at the three time points investigated (5, 12, and 19 days after the start of treatment; (Figure 3A–3C)), the strongest effect being observed at day 19. Surprisingly, similar results were obtained when DTIC was administered alone, suggesting an effect of the cytotoxic agent on MAPK pathway activation. The combination of selumetinib + DTIC induced a decrease in p-ERK expression that was particularly pronounced 19 days after the start of treatment. By contrast, we observed an increase in p-MEK levels after selumetinib administration, either alone or in combination with DTIC, at the three time points studied, consistent with the regulation of MEK1/2 inhibitor activity by a negative control loop. Finally, Pi3K pathway revealed a slight decrease in p-S6 expression after DTIC treatment (Figure 3B–3D).

**Figure 4:**
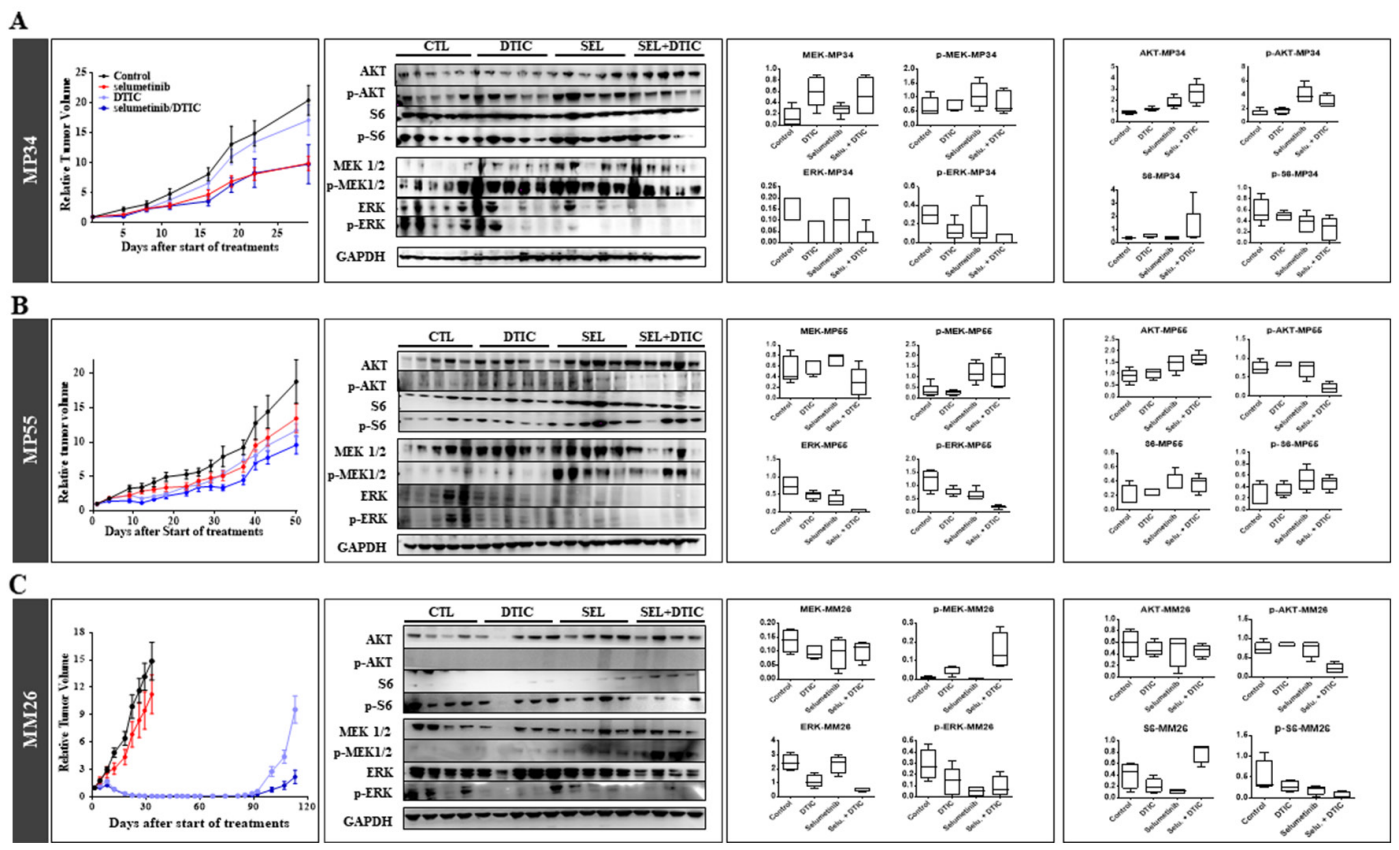
Pharmacodynamics (PD) study of the combination of selumetinib and DTIC in the three treated UM PDXs **(A)** MP34. **(B)** MP55. **(C)** MM26. For each model, *in vivo* efficacy is shown, with western blot and normalized MAPK (MEK, p-MEK, ERK, p-ERK) and Pi3K (AKT, p-AKT, S6, p-S6) protein levels determined at the end of *in vivo* experiments.

A PD study on tumors collected at the end of *in vivo* experiments on the three UM PDXs, MP34 (Figure 4A), MP55 (Figure 4B), and MM26 (Figure 4C), showed that both selumetinib and DTIC reduced p-ERK expression when used separately, but that the decrease was larger when these treatments were combined. As previously observed in the MP55 model, we observed an increase in p-MEK expression for all therapeutic schedules, in both MP55 and MM26 PDXs, but not in the MP34 xenograft. By contrast, none of the treatments tested had any effect on the Pi3K signaling pathway.

These data validate the PD activity of selumetinib administered alone, resulting in a decrease in p-ERK and an increase in pMEK expression, and they reveal a similar effect of DTIC alone and of selumetinib + DTIC. Given the small number of PDXs tested, it was not possible to correlate PD observations with efficacy *in vivo*.

### *In vivo* efficacy of several selumetinib-based combinations

**Figure 5:**
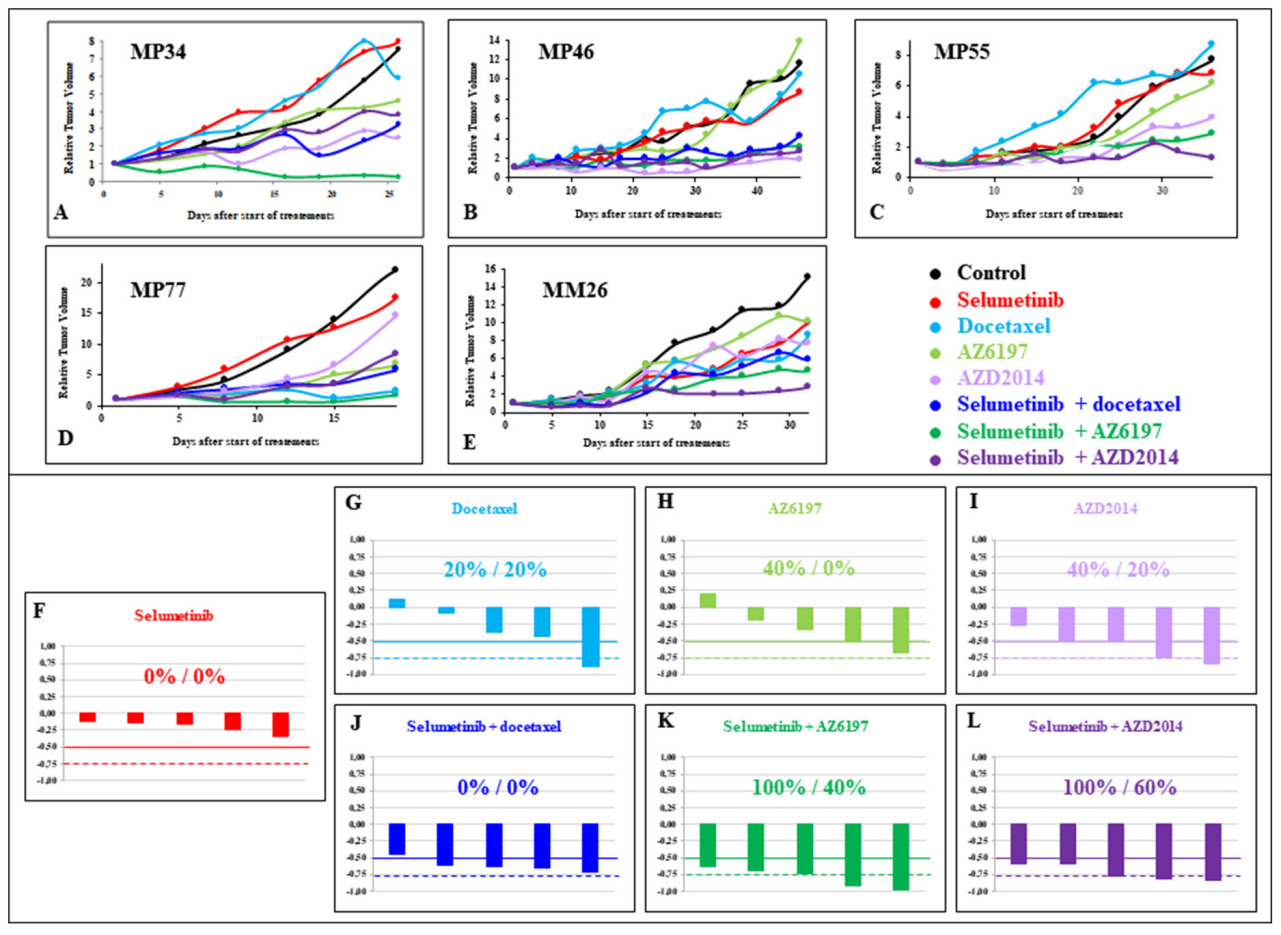
*In vivo* efficacy of new selumetinib-based combinations Tumor growth inhibition due to selumetinib with and without docetaxel, AZ6197 and AZD2014 in the five UM PDXs, MP34 **(A)**, MP46 **(B)**, and MP55 **(C)**, MP77 **(D)**, and MM26 **(E)**. Tumor growth was evaluated by plotting RTV (relative tumor volume) par mouse. Overall responses, representing the response of an individual mouse to selumetinib **(F)**, docetaxel **(G)**, AZ6197 **(H)**, AZD2014 **(I)**, selumetinib + docetaxel **(J)**, selumetinib + AZ6197 **(K)**, and selumetinib + AZD2014 **(L)**.

The efficacy of three other combinations with selumetinib that have yet to be tested clinically was evaluated in five UM PDXs (MP34, MP46, MP55, MP77, and MM26). The drugs used in combination with selumetinib were docetaxel, the ERK inhibitor AZ6197, and the mTORC1/2 inhibitor AZD2014. Monotherapies were used as controls in all experiments with combinations. The data are presented in Figure 5, Supplementary Figure 2, and Supplementary Tables 1, 2, and 3.

Monotherapies yielded TGI values of 11% to 34% for selumetinib, 0% to 88% for docetaxel, 0% to 67% for AZ6197, and 28% to 84% for AZD2014. Treatment with selumetinib did not significantly decrease (less than -0.5) the ORR in any of the PDX models. An ORR below 0.5 was observed in one model with docetaxel, two with AZ6197 and two with AZD2014 (Supplementary Table 1). For the establishment of an efficacy-based classification of the four monotherapies tested, we ranked all the tested agents in terms of their efficacy, according to a method based on TGI criteria (see Materials and Methods). The results are shown in Supplementary Table 2. Selumetinib, docetaxel and AZ6197 scored 16, 14, and 13, respectively, and the total score for AZD2014 was 7, highlighting the greater efficacy of this mTORC1/2 inhibitor than of the other compounds.

In analyses of combinations, TGI was 45% to 71% for selumetinib + docetaxel, 62% to 97% for selumetinib + AZ6197, and 59% to 83% after selumetinib + AZD2014. An ORR below -0.5 was obtained for four of the five PDXs following treatment with selumetinib + docetaxel, and in all five models for both selumetinib + AZ6197 and selumetinib + AZD2014 (Supplementary Table 3). The final scores for selumetinib + docetaxel, selumetinib + AZ6197 and selumetinib + AZD2014 were 13, 8, and 9, respectively. When the score was calculated for all seven tested treatments (monotherapies and treatment combinations), the best two treatments were selumetinib + AZ6197 and selumetinib + AZD2014, with scores of 9 and 13, respectively (Supplementary Table 2).

Finally, the combination treatments resulted in a slight increase in ORR (< -0.5) for selumetinib + docetaxel relative to selumetinib or docetaxel alone, and a significant increase for both selumetinib + AZ6197 and selumetinib + AZD2014 relative to monotherapies (Supplementary Figure 2).

### Pharmacodynamic study of selumetinib-based combinations

**Figure 6:**
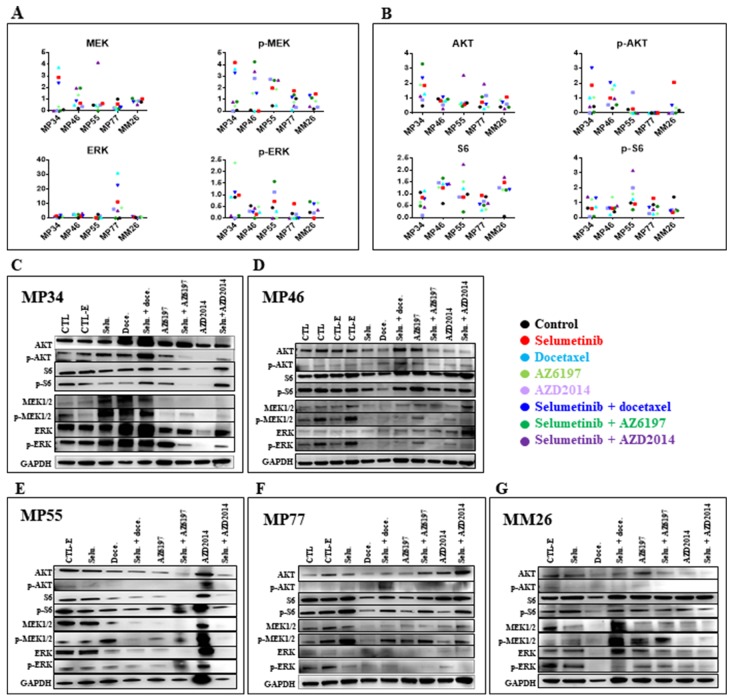
Pharmacodynamics (PD) study of new selumetinib-based combinations Normalized MAPK (MEK, p-MEK, ERK, p-ERK) **(A)** and Pi3K (AKT, p-AKT, S6, p-S6) **(B)** protein levels determined at the end of *in vivo* experiments. Western blot of MAPK (MEK, p-MEK, ERK, p-ERK) and Pi3K (AKT, p-AKT, S6, p-S6) protein levels (AKT, p-AKT, S6, p-S6) in the MP34 **(C)**, MP46 **(D)**, MP55 **(E)**, MP77 **(F)**, and MM26 **(G)** UM PDXs.

**Figure 7:**
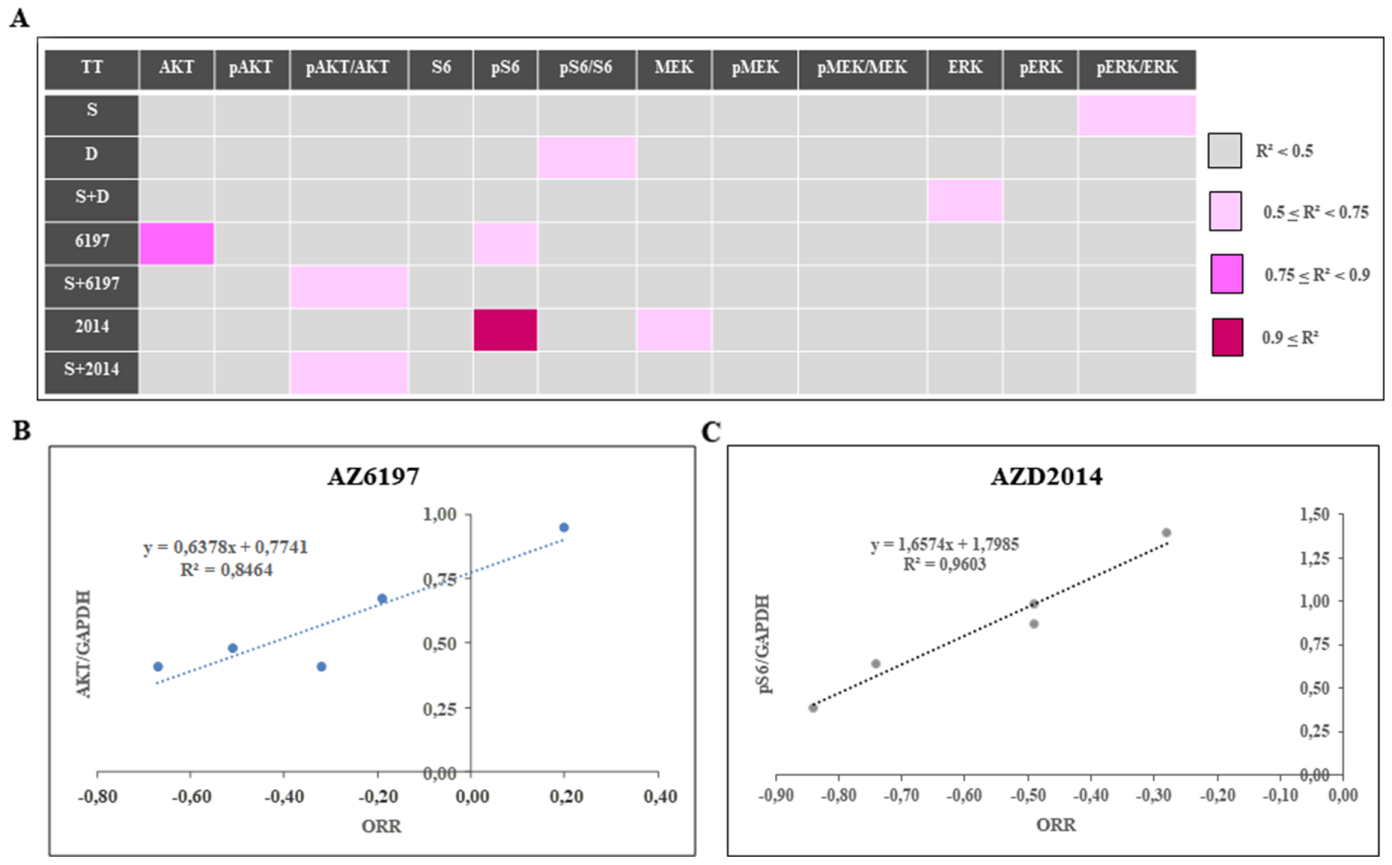
Determination of predictive markers of the response to new selumetinib-based combinations **(A)** Study of the overall correlation between mean tumor growth inhibition for a given PDX and the individual normalized protein level in the control group, in an r^2^ test (Spearman test). Correlations were classified into four groups: weak correlation if r^2^ < 0.5, weak-intermediate if 0.5 ≤ r^2^ < 0.75, intermediate-strong if 0.75 ≤ r^2^ < 0.9, and strong if 0.9 ≤ r^2^. **(B)** Correlation betweenAKT levels and the response to AZ6197. **(C)** Correlation between p-S6 levels and the response to AZD2014.

A PD study was performed on tumors collected at the end of *in vivo* experiments on the five treated UM PDXs, MP34, MP46, MP55, MP77, and MM26 (Figure 6). The principal modifications to the MAPK pathway (Figure 6A) observed concerned p-ERK expression, which was increased by the administration of AZ6197 and decreased by the administration of AZD2014, either alone or in combination with selumetinib. For the Pi3K signaling pathway, we observed an increase in p-AKT and/or S6 expression in some of the PDX models. We were able to establish two significant correlations, between AKT expression and response to AZ6197 (Figure 7A, 7B), and between p-S6 expression and response to AZD2014 (Figure 7A, 7C). The potential use of the levels of these proteins as biomarkers predictive of response would require validation in a larger number of models.

## DISCUSSION

In this study, we used UM PDXs to evaluate the efficacy of the MEK inhibitor selumetinib, alone or in combination with cytotoxic and targeted therapies. The ultimate goal of this work is the development of clinical strategies for improving the response to selumetinib in patients with UM. We first performed a preclinical study mimicking the study design and rationale of a randomized, placebo-controlled, double-blind protocol assessing the efficacy of selumetinib (AZD6244; ARRY-142886) in combination with dacarbazine in patients with metastatic uveal melanoma (SUMIT). [11]

Our preclinical study showed that selumetinib had only weak efficacy *in vivo* when used alone, with an overall response rate of 18% and no tumor shrinkage or complete remission. These data are similar to those obtained in the clinical trial, in which 14% of patients achieved an objective radiographic response to treatment [10]. Progression-free-survival and response rate we for selumetinib compared to TMZ and indicates that drug combinations are required to improve the clinical benefit of MEK inhibition. In our *in vitro* preclinical assays, we have observed a trend towards a synergistic activity between selumetinib and DTIC in uveal melanoma cell lines; in contrast, these results did not translate to the *in vivo* experiments using UM PDXs where the ORR was non-significantly increased by combining selumetinib and DTIC in comparison with when compared with the agents administrated alone. SUMIT clinical trial [11] where the DTIC treatment. There was a strong correlation between preclinical and clinical effects in our UM PDXs. Our pharmacokinetic studies clearly showed that the observed lack of improvement in ORR was not due to the modification of selumetinib pharmacokinetics by concomitant DTIC administration. Given the small number of UM PDXs treated, it was not possible to demonstrate a correlation between response and the genetic background of the models. Indeed, all the PDXs tested had *GNAQ*/*11* mutations, but only one had a *BAP1* mutation and two had *SF3B1*mutations. Thus, all the preclinical and clinical results suggest that, despite the presence of *GNAQ* or *GNA11* mutations, targeting MEK activity is not sufficient for the effective treatment of metastatic UM. New combination strategies are required and should be evaluated.

To this end, we tested new selumetinib-based combinations in our UM PDXs: selumetinib + docetaxel, selumetinib + the ERK inhibitor AZ6197, and selumetinib + the mTORC1/2 inhibitor AZD2014. In this part of the study, we treated one mouse per group, according to the strategy recently described by Gao and colleagues [19]. The experiments were performed using five different UM PDXs. We observed no response to selumetinib alone, but a response to docetaxel was observed in one of our models. No preclinical or clinical data concerning the efficacy of docetaxel as a monotherapy for UM have ever been reported. A phase II study of docetaxel + carboplatin has been reported, in which disease stabilization was the best result obtained.

In our preclinical studies, AZ6197 and AZD2014 displayed significant efficacy in two PDXs. These results provide the first evidence for the efficacy of ERK inhibition for treating uveal melanoma. We have already shown mTORC1 inhibitors to be effective, in both UM cell lines and PDXs [15, 20]. With our scoring method, AZD2014 was found to be the most effective of the agents evaluated in monotherapy. *In vivo* evaluations of selumetinib-based combinations showed that selumetinib + AZ6197 and selumetinib + AZD2014 were the best combination treatment strategies. Our data therefore suggest that approaches targeting ERK and mTORC1/2 are promising for the treatment of metastatic UM.

Our *in vivo* experiments indicate that the clinical feasibility of such combinations remains an issue, because it is not possible to evaluate the potential toxicity of such combinations in patients. The toxicity of combinations, requiring dose modifications, has been observed with MEK inhibitors and other targeted agents [21]. We can conclude from our study that there is a strong rationale for the use of selumetinib in combination with the ERK inhibitor AZ6197 or the mTORC1/2 inhibitor AZD2014 in patients with metastatic uveal melanoma. As discussed by Decaudin and Le Tourneau, this preclinical work is a prerequisite for subsequent clinical trials.

## MATERIALS AND METHODS

### Compounds

Two chemotherapy agents, the alkylating agent dacarbazine (DTIC) (Medac, France) and the taxane docetaxel (Sanofi-Aventis, France), and three targeted drugs, the MEK1/2 inhibitor selumetinib, the ERK inhibitor compound 35 (also known as AZ6197) [22], and the mTORC1/2 inhibitor AZD2014, were used in this study. All targeted therapies were provided by Astra Zeneca (Oncology Bioscience, Cambridge, UK).

*In vitro* experiments were performed with selumetinib and DTIC. Both compounds were dissolved in DMSO to generate 10 mM stock concentrations, which were dispensed into aliquots and stored at -20°C.

For *in vivo* experiments, selumetinib was suspended in 0.5% *v/v* Tween 80 and 0.5% methylcellulose and administered orally at a dose of 25 mg/kg, BID, 5 days/week. AZ6197 was dissolved in 10% DMSO, made up to the required volume with 40% hydroxypropyl β cyclodextrin (HPCD) and administered orally at a dose of 50 mg/kg, QD, 5 days/week. AZD2014 was diluted in 1% polysorbate and administered orally at a dose of 15 mg/kg, QD, 5 days/week. Dacarbazine (DTIC) was administered at a dose of 40 mg/kg, for five consecutive days, every 28 days. Docetaxel (DOC, Taxotere, Sanofi Aventis) was administered weekly at a dose of 15 mg/kg. All cytotoxic drugs were reconstituted in 0.9% NaCl and administered by intraperitoneal (IP) injection.

### Uveal melanoma cell lines

Six uveal melanoma cell lines established in our laboratory [13] were used for *in vitro* experiments. They were obtained from primary tumors (MP38, MP41, MP46, and MP65), or from metastases from patients (MM66 and MM28). All cell lines were established from PDXs, with the exception of MP38 and MP65, which were obtained directly from patient tumor samples. Short tandem repeat polymorphism (STR) analysis was performed to confirm that all the cell lines matched the tumor of origin [13].

These cell lines were maintained as monolayers in RPMI supplemented with 20% fetal bovine serum, 100 U/mL penicillin and 100 μg/mL streptomycin, and incubated at 37°C under an atmosphere containing 5% CO_2_. The molecular features of the six cell lines are presented in Supplementary Table 4.

### Uveal melanoma PDXs

Five UM PDXs were used in this study: MP34, MP46, MP55, MP77, and MM26. The molecular features of these models are presented in Supplementary Table 5.

### *In vitro* cell viability analyses

Cell viability was determined in a colorimetric MTT [3-(4,5-dimethylthiazol-2-yl)-2,5-diphenyl-2H tetrazolium bromide] bioassay (Sigma). The cells were plated in 96-well plates at an appropriate density (Supplementary Table 6), in triplicate. They were incubated overnight and treated with various concentrations of DTIC or selumetinib for 72 h. Cell viability was assessed in MTT bioassays, as follows: briefly, the drug-containing medium was removed and replaced with 100 μL of medium supplemented with 20% MTT. The cells were incubated for 4 h at 37°C. We then added 100 μL of 10% SDS/10 mM HCl to each well. Absorbance was measured at 570 and 620 nm. Cell viability was evaluated by determining ΔOD (570- 620 nm) and was normalized against the ΔOD (570- 620 nm) of control cells.

### Evaluation of cell viability assays for the various drug combinations

Combination treatments were administered with serial dilutions of DTIC and selumetinib in a 1:2 ratio, for all six uveal melanoma cell lines. This ratio was based on the IC_50_ values obtained for these drugs in the initial experiments. Cells were used to seed three 96-well plates at appropriate densities, according to a 6x6 matrix design. The following day, the drugs were added according to a matrix dilution format. Cell viability was measured after three days of drug treatment, with the MTT assay, and expressed as the percentage of metabolically inactive cells relative to the DMSO-treated control (percent growth inhibition). We assessed the synergistic effect of combinations of selumetinib and DTIC, as previously described [23].

### *In vivo* tumor growth and antitumor efficacy

For *in vivo* therapeutic studies, a 20-40 mm^3^ tumor fragment was xenografted into female SCID mice (Janvier Laboratories, France). Mice bearing growing tumors with a volume of 60-150 mm^3^ were randomly assigned to the control or treatment groups (the number of animal per group is detailed in the figure legends). Animals with tumor volumes outside this range were excluded. Treatments were started on day 1. Mice were weighed and tumors were measured twice weekly. Xenografted mice were killed when tumor volume reached 2500 mm^3^. Tumor volume (V) was calculated by measuring two perpendicular diameters with calipers and applying the following formula: V = *a* × *b*^2^ / 2, where *a* and *b* are the largest and smallest perpendicular tumor diameters. Relative tumor volume (RTV) was calculated as follows: RTV = (Vx/V1), where Vx is the tumor volume on day x and V1 is the tumor volume at the start of treatment (day 1). Growth curves were obtained by plotting the mean values of RTV on the *y* axis against time on the *x* axis, expressed as days after the start of treatment. Antitumor activity was evaluated by determining tumor growth inhibition (TGI) as follows: percent GI = 100 − (RTVt / RTVc × 100), where RTVt is the median RTV of treated mice and RTVc is the median RTV of controls, both for the time point at which antitumor effect was optimal. A meaningful biological effect was defined as a TGI of at least 50%. The statistical significance of the differences observed between the individual RTVs corresponding to the treated and control mice was determined in two-tailed Mann-Whitney tests.

We evaluated treatment responses in all treated models as a function of individual mouse variability, by considering each mouse as a single tumor-bearing entity. Hence, in all *in vivo* experiments, a relative tumor volume variation (RTVV) was calculated for each treated mouse as follows: [(RTVt/mRTVc)], where RTVt is the relative tumor volume of the treated mouse and mRTVc is the median relative tumor volume of the corresponding control group at the end of treatment.

We then calculated [(RTVV)-1] for each treated mouse. A tumor was considered to be responding to treatment if [(RTVV)-1] was below -0.5. Finally, we evaluated the impact of treatments on tumor progression, by evaluating all progression-free survival probabilities taking tumor doubling time into account [19].

These studies were performed in accordance with the recommendations of the French National Ethics Committee and under the supervision of authorized investigators. The experimental protocol and animal housing complied the institutional guidelines laid down by the French National Ethics Committee (agreement number D-750602, France) and the ethics committee of Institut Curie (agreement number C75-05-18). In addition, all animal experiments were submitted to AstraZeneca for internal ethical review.

### Pharmacokinetic study of selumetinib alone or in combination with DTIC

For the pharmacokinetic study, three SCID mice without grafts were orally treated with selumetinib at a dose of 25 mg/kg, BID, for five days. On day 6 they were treated once and 20 μL of blood was collected into a coated heparin capillary and mixed with 80 μL phosphate buffer, 0.5, 2, 4 and 8 hours after the last selumetinib administration. In studies of combinations, we also administered DTIC on five consecutive days, at a dose of 40 mg/kg IP. Blood diluted in PBS was centrifuged at 16200 x *g* for 3minutes at 4°C, and plasma was collected and stored at -80°C.

Each plasma sample (25 μL) was prepared using an appropriate dilution factor, and compared with an 11-point standard calibration curve (1-10000 nM) prepared by spiking blank plasma samples with stock solution in DMSO. Acetonitrile (100 μL) was added with the internal standard, and the mixture was centrifuged at 3000 rpm for 10 minutes. Supernatant (50 μL) was then diluted in 300 μL water and analyzed by UPLC-MS/MS (Supplementary Tables 7 and 8).

### Western blotting and the pharmacodynamic biomarker study

Proteins were extracted as previously described [24]. Lysates were resolved by electrophoresis in 4–12% TGX gels (Bio-Rad^®^), and the resulting bands were transferred onto nitrocellulose membranes (Bio-Rad^®^) and probed with rabbit antibodies against GAPDH (Cell Signaling Technology, #2118), P-p44/42 MAPK (Thr202/Tyr204) (Cell Signaling Technology, #4370), p44/42 MAPK (Cell Signaling Technology, #9102), S6 (Cell Signaling Technology, #2117), P-S6 (Ser235/236) (Cell Signaling Technology, #2211), MEK1/2 (Cell Signaling Technology, #9126), p-MEK1/2 ser217/221) (Cell Signaling Technology, #9154), AKT (Cell Signaling Technology, #9272) and P-AKT (ser473) (Cell Signaling Technology, #4058). The membranes were washed and incubated with the appropriate horseradish peroxidase-conjugated affinity-purified goat anti–rabbit secondary antibody (Jackson Immuno Research Laboratories, Inc., Interchim). P-MEK1/2, MEK1/2, P-S6, S6, P-p44/42 MAPK, p44/42MAPK, AKT and P-AKT were quantified with Multi Gauge software and normalized against GAPDH levels. For each PDX model, the ratio of P-MEK1/2 / MEK1/2 in selumetinib-treated tumors *versus* control tumors was calculated as follows: mean P-MEK/MEK level in three selumetinib-treated xenografts/mean P-MEK/MEK level in four control xenografts. The same method was used to quantify P-S6/S6, P-p44/42MAPK/p44/42MAPK and P-AKT/AKT. The differences between responder, weak responder and resistant xenografts was analyzed by one-way ANOVA.

Predictive biomarkers were identified on the basis of correlations between the mean tumor growth inhibition of the PDX and the individual protein value normalized relative to the control group in an r^2^ test (Spearman test). Correlations were classified into four groups: weak correlation if r^2^ < 0.5, weak-intermediate correlation if 0.5 ≤ r^2^ < 0.75, intermediate-strong correlation of 0.75 ≤ r^2^ < 0.9, and strong correlation if r^2^ ≥ 0.9.

## SUPPLEMENTARY MATERIALS FIGURES AND TABLES



## Abbreviations

PDX: Patient Derived Xenograft
UM: uveal melanoma
MAPK: mitogen-activated protein kinase
GNAQ/GNA11: Guanine nucleotide-binding protein G(q) subunit alpha
MEK: mitogen-activated protein kinase kinase
MEKi: MEK inhibitor
ERK: extracellular regulating kinase
mTORC1/2: mammalian target of rapamycin complex 1/2
Pi3K: phosphoinositide 3-kinase
PKC: protein kinase C
MDM2: murine double minute 2
c-MET: tyrosine-protein kinase Met
IP: intraperitoneal
PO: *per os* (gavage)
RTV: relative tumor volume
DTIC: dacarbazine
DOC: docetaxel
PD: pharmacodynamic
PK: pharmacokinetic
TGI: tumor growth inhibition
ORR: overall response rate
MTT: [3-(4,5-dimethylthiazol-2-yl)-2,5-diphenyl-2H tetrazolium bromide]
CR: complete response
HPCD: hydroxypropyl β cyclodextrin
