# Supplementary material for: Selumetinib-based therapy in uveal melanoma patient-derived xenografts
Source: Oncotarget. 2018 Apr 24;9(31):21674–86. doi: 10.18632/oncotarget.24670 (PMC5955168; doi:10.18632/oncotarget.24670)
Supplement: Supplementary file 1 [file oncotarget-09-21674-s001.pdf]

Selumetinib-based therapy in uveal melanoma patient-derived xenografts

SUPPLEMENTARY MATERIALS

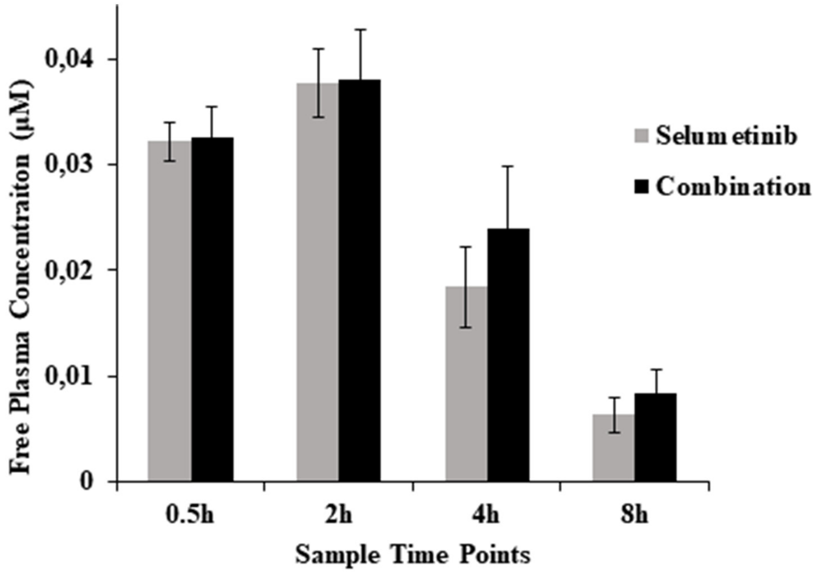

Supplementary Figure 1: *In vivo* pharmacokinetics of selumetinib administered alone or in combination with DTIC.

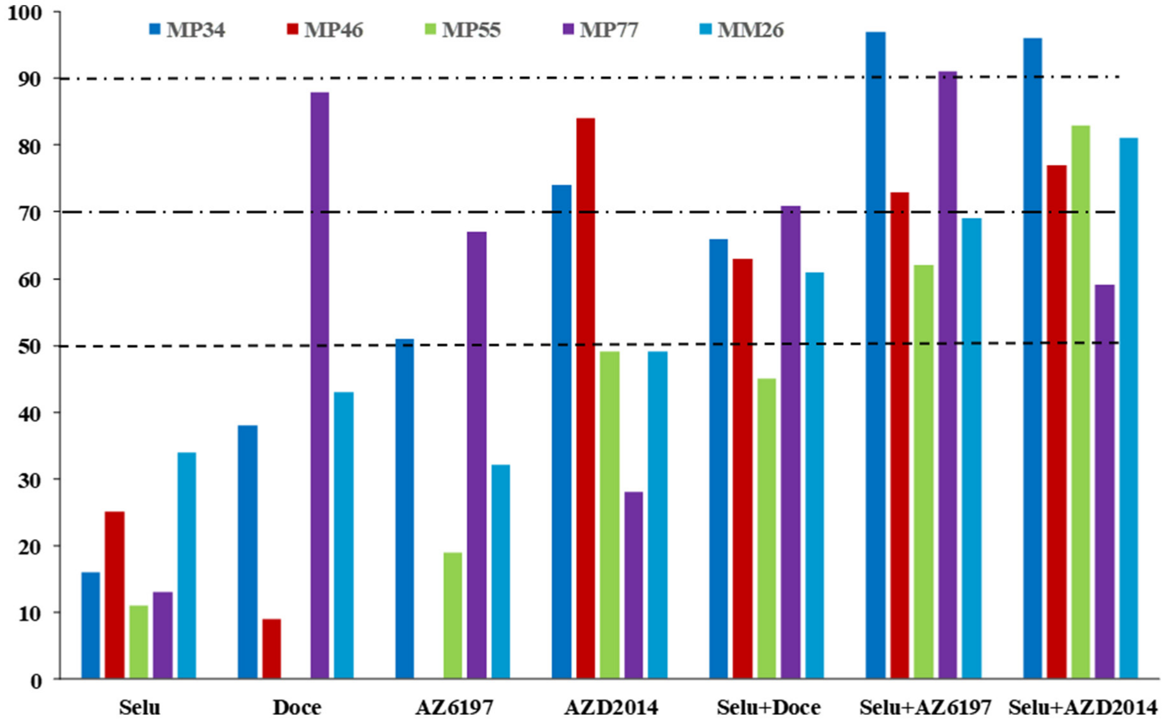

Supplementary Figure 2: Overall responses to new selumetinib-based combinations.

Supplementary Table 1: ***In vivo*** **efficacy of targeted therapies administered alone**

**All PDXs (%)**	**Treatments**			
	**Selumetinib**	**Docetaxel**	**AZ6197**	**AZD2014**
**RTV ≤ 1**	0	0	0	0
**ORR ≤ -0.5**	0	20	40	40
**ORR ≤ -0.75**	0	20	0	20
**mPFS (days)**	11	10	12	23

Abbreviations: RTV, Relative Tumor Volume; ORR, Overall Response Rate; mPFS, median of progression-free survival.

Supplementary Table 2: *In vivo* efficacy score for targeted therapies administered alone or in combinations with Selumetinib

Treatments		MP42	MP46	MP55	MP77	MM26	Total
**4 monotherapies**	**Selumetinib**	4	2	3	4	3	**16**
**(score 1 to 4)**	**Docetaxel**	3	4	4	1	2	**14**
	**AZ6197**	2	3	2	2	4	**13**
	**AZD2014**	1	1	1	3	1	**7**
**3 combinations**	**S + D**	2	3	3	2	3	**13**
**(score 1 to 3)**	**S + AZ6197**	1	2	2	1	2	**8**
	**S + AZD2014**	3	1	1	3	1	**9**
**7 treatments**	**Selumetinib**	7	5	6	7	6	**31**
**(score 1 to 7)**	**Docetaxel**	6	6	7	2	5	**26**
	**AZ6197**	5	7	5	4	7	**28**
	**AZD2014**	2	1	3	6	4	**16**
	**S + D**	3	4	4	3	3	**17**
	**S + AZ6197**	1	3	2	1	2	**9**
	**S + AZD2014**	4	2	1	5	1	**13**

Abbreviations: S, selumetinib; D, docetaxel.

Supplementary Table 3: *In vivo* efficacy of selumetinib-based combinations

All PDXs (%)	Treatments		
	**S + D**	**S + AZ6197**	**S + AZD2014**
**RTV ≤ 1**	0	20	0
**ORR ≤ -0.5**	80	100	100
**ORR ≤ -0.75**	0	40	60
**mPFS (days)**	16	22	16

Supplementary Table 4: Characteristics of UM cell lines

Cell lines	Tumor sample origin	Morphology	LOH of chromosome 3	*BAP1* mutations	BAP1 Protein production[3]	*GNAQ* mutations	*GNA11* mutations	*SF3B1* Mutations
**MP38**	**Patient (eye)**	S	Yes[1]	Yes	No	Yes	No	No
**MP41**	**PDX (eye)**	E	Yes[2]	No	Yes	No	Yes	No
**MP46**	**PDX (eye)**	M	Yes	No	No	Yes	No	No
**MP65**	**Patient (eye)**	S	Yes	Yes	No	No	Yes	No
**MM28**	**PDX (liver)**	M	Yes[1]	Yes	No	No	Yes	No
**MM66**	**PDX (liver)**	M	No	No	Yes	No	Yes	No

Abbreviations: S, spindle cells; M, mixed cells; E, epithelioid cells.
[1]Uniparental disomy of 3q.
[2] Uniparental disomy of chromosome 3.
[3] as determined by Western blotting and Immunohistochemistry.

Supplementary Table 5: Characteristics of UM PDXs

Cell lines	Tumor sample origin	Morphology	LOH of chromosome 3	*BAP1* mutations	BAP1 Protein production[3]	*GNAQ* mutations	*GNA11* mutations	*SF3B1* Mutations
**MP34**	**Eye**	E	Yes[1]	No	Yes	No	Yes	Yes
**MP46**	**Eye**	M	Yes	No[3]	No	Yes	No	No
**MP55**	**Eye**	E	Yes	Yes	No	Yes	No	No
**MP77**	**Eye**	E	Yes	No	Yes	No	Yes	No
**MM26**	**Liver**	E	Yes	No	Yes	Yes	No	Yes

Abbreviations: S, spindle cells; M, mixed cells; E, epithelioid cells.
[1]Uniparental disomy of 3q.
[2] As determined by Western blotting and Immunohistochemistry.
[3] Mutation in promoter.

Supplementary Table 6: Density of cell lines in viability tests *in vitro*

Cell lines	number of cells/well
**MP38**	8000
**MP65**	8000
**xMP41**	2000
**xMP46**	7000
**xMM28**	5000
**xMM66**	6000

Supplementary Table 7: Mass Spectrometer and UPLC system parameters

**Mass Spectrometer**	Waters Xevo TQS		
**UPLC system**	Water s Acquity i-Class		
**Column**	Phenomenex Kinetix C18 50 x 2.1 x 1.7 µm		
**Solvent A**	95% (*v/v*) Water, 5% Methanol + 0.1% Formic acid		
**Solvent B**	95% MeOH, 5% Water + 0.1 % Formic acid		
**Elution Gradient**	Time (min)	**% A**	**%B**
	0	95	5
	0.3	95	5
	2.2	5	95
	2.6	5	95
	2.61	95	5
	2.8	95	5
**Flow**	0.6 mL/min		
**Run time**	2.8 min, use a divert valve for initial 0.5 minutes		

Supplementary Table 8: Optimization parameters for mass spectrometry analysis

Compound	Ionization mode	Polarity	Parent ion	Daughter ion	Cone voltage (v)	Collison Energy	Retention Time (min)
Selumetinib	ESI	Positive	459.155	396.846	60	22	1.71
AZ10024306	ESI	Positive	408.064	174.241	60	22	1.53
